# 

**Published:** 1913-02-22

**Authors:** 


### Gifts and Bequests.

Mr. John Bellamy, of Harborne, Birmingham, retired chemist and druggist, has left £1,500 among a number of Birmingham charities and £100 to the Benevolent Fund of the Pharmaceutical Society.

Miss Jean Hope has bequeathed £1,000 to the Middlesex Hospital, for the endowment of a bed in the names of Robert Hope and Mary Hope, her parents; £1,250 to the Dumfries and Galloway Royal Infirmary, for the endowment of a bed in the names of Robert Hope, sen., and Robert Hope, jun.; £500 to the East End Mothers' Lying-in Home; and some smaller legacies to hospitals.

Miss Theresa Jane Wheler, of Westbourne Gardens, Bayswater, who died on December 8, left estate valued at £14,858 gross, with net personalty £14,640. She bequeathed £200 to the Metropolitan Drinking Fountain and Cattle Trough Association, £100 to the Union Jack Club for a "Theresa Jane Wheler" bedroom, and, subject to a life interest, £1,000 to the Royal Society for the Prevention of Cruelty to Animals "for prosecutions in all cases of cruelty to cats," and £500 each to the Royal Sailors' Rests at Portsmouth and the Royal Sailors' Rests at Devonport.

### Doctors' Wills.

Mr. Francis John Loveday, M.R.C.S., L.R.C.P., of Bedford Park, medical referee to the Hearts of Oak Assurance Company, left estate of the value of £43,169 gross and £14,244 net.

The annual general meeting of the Governors of the Cancer Hospital (Free), Fulham Road, S.W., will be held on Wednesday, February 26, 1913, at 4 p.m., the Right Hon. the Earl of Northbrook, president, in the chair.

Messrs. J. and A. Churchill are about to publish an important new book entitled "The Difficulties and Emergencies of Obstetric Practice," by Mr. Comyns Berkeley and Mr. Victor Bonney, obstetric and gynæcological surgeons to the Middlesex Hospital. The illustrations are all original and very numerous. They are, moreover, the design of an expert artist, who is himself a medical man. The same publishers have just ready the sixth edition of "A Short Practice of Midwifery," by Dr. Jellett, master of the Rotunda Hospital, Dublin. The new edition is to be issued with a larger page, and has been revised throughout and contains many new illustrations. The popularity of the book is evident by the fact that 20,000 copies have been printed.

**RATEPAYERS! POLL IN SELF-DEFENCE.**

**The present Council election should result in a much larger poll than ever before, because no single vote should be given at the ensuing election for any candidate of either party who is not pledged, if elected, to protect the citizens without a moment's further delay from the pressing dangers and miseries to which every one of them is daily subjected through the past neglect to supply an efficient Ambulance Service, by the retiring members of all parties of the present L.C.C. It is the London County Councillors' duty to establish an efficient ambulance service, and they must not be permitted to shirk this duty but urged to undertake it at once Otherwise government by County Council, so far as London is concerned, may be shown to be little if any better than that of the old Metropolitan Board of Works.**

### Appointments.

Mr. J. F. O'Connell has been appointed fifth assistant medical officer at Hanwell Mental Hospital.

Mr. D. T. O'Flynn has been promoted to be fourth assistant medical officer at Cane Hill Mental Hospital.

Mr. E. E. Isaac has been promoted to the position of third assistant medical officer at Colney Hatch Mental Hospital.

The annual court of the Corporation of King's College Hospital will be held in the Board Room on Thursday, the 27th inst., at five o'clock.

Sir William Collins's lecture on the 17th inst to the members of the Medical Graduates' College and Polyclinic, entitled "An Efficient Ambulance Service for Accident and other Casualties in Streets and Public Places in London," will be published in full, we understand, in the *Medical Press and Circular*.

The annual meeting of the After Care Association for Poor Persons Discharged Recovered from Asylums for the Insane will be held at the Medical Society's Rooms, 11 Chandos Street, Cavendish Square, W., on Tuesday, February 25, 1913, at 2.45 p.m., Sir James Moody presiding. After the usual business a paper, entitled "After Care in Cases of Mental Disorder and the Desirability of Extending its Scope," will be read by Dr. Hubert Bond (Commissioner in Lunacy), to be followed by a discussion.

### Manager's Notices.

**Letters relating to the publication, sale, and advertising departments of "The Hospital" must be addressed to The Manager, "The Hospital," The Hospital Building, 28 and 29 Southampton Street, London, W.C.**

**Subscriptions** may begin at any time, and are payable in advance. Cheques and Post-Office Orders should be crossed London County and Westminster Bank, Covent Garden Branch, and made payable to the Manager of The Hospital.

Rates of Subscription (including Postage).

| *United Kingdom.* | | *Foreign and Colonial.* | |
|---|---|---|---|
| Three Months ... ... | 2s. 0d. | Three Months ... ... | 2s. 6d. |
| Six Months ... ... | 4s. 0d | Six Months ... ... | 5s. 0d. |
| Twelve Months ... ... | 6s. 6d. | Twelve Months ... ... | 8s. 8d. |

**Advertisements:**

To ensure insertion, all advertisements for The Hospital must reach the Manager not later than Wednesday morning in each week. For scale of charges see pages iii and 4.

